# Artificial Intelligence and Healthcare Simulation: The Shifting Landscape of Medical Education

**DOI:** 10.7759/cureus.59747

**Published:** 2024-05-06

**Authors:** Allan Hamilton

**Affiliations:** 1 Artificial Intelligence Division, Arizona Simulation Technology and Education Center (ASTEC) University of Arizona, Tucson, USA

**Keywords:** neural blockade, deep learning, machine learning, task trainers, manikin, simulation, artificial intelligence

## Abstract

The impact of artificial intelligence (AI) will be felt not only in the arena of patient care and deliverable therapies but will also be uniquely disruptive in medical education and healthcare simulation (HCS), in particular. As HCS is intertwined with computer technology, it offers opportunities for rapid scalability with AI and, therefore, will be the most practical place to test new AI applications. This will ensure the acquisition of AI literacy for graduates from the country’s various healthcare professional schools. Artificial intelligence has proven to be a useful adjunct in developing interprofessional education and team and leadership skills assessments. Outcome-driven medical simulation has been extensively used to train students in image-centric disciplines such as radiology, ultrasound, echocardiography, and pathology. Allowing students and trainees in healthcare to first apply diagnostic decision support systems (DDSS) under simulated conditions leads to improved diagnostic accuracy, enhanced communication with patients, safer triage decisions, and improved outcomes from rapid response teams. However, the issue of bias, hallucinations, and the uncertainty of emergent properties may undermine the faith of healthcare professionals as they see AI systems deployed in the clinical setting and participating in diagnostic judgments. Also, the demands of ensuring AI literacy in our healthcare professional curricula will place burdens on simulation assets and faculty to adapt to a rapidly changing technological landscape. Nevertheless, the introduction of AI will place increased emphasis on virtual reality platforms, thereby improving the availability of self-directed learning and making it available 24/7, along with uniquely personalized evaluations and customized coaching. Yet, caution must be exercised concerning AI, especially as society’s earlier, delayed, and muted responses to the inherent dangers of social media raise serious questions about whether the American government and its citizenry can anticipate the security and privacy guardrails that need to be in place to protect our healthcare practitioners, medical students, and patients.

## Introduction and background

Artificial intelligence (AI) is revolutionizing the field of healthcare education with a transformative power reminiscent of the historical impact delivered by the Flexner Report on American Medical Education in 1910 [1]. Originally commissioned by the Carnegie Foundation, the report radically altered medical education and academic medicine in the United States. Some of the most radical, accelerated changes that will be brought about by AI will occur in the field of healthcare simulation (HCS) because, first, the realm of simulation-based training is so deeply enmeshed with computer technology that it makes an HCS an exceptionally fertile ground for AI integration [2]. Second, as we will explore, HCS is uniquely positioned for the rapid scalability of AI, offering unparalleled opportunities to use and evaluate its impact. Third, medical education, with its focus on both teaching and assessing the power of AI in healthcare, will increasingly rely on HCS as AI’s training and proving ground. These unique demands will place a significant responsibility on HCS to evolve and adapt swiftly. Finally, and perhaps most crucially, the shift towards AI-compatible modalities and delivery systems offers both methodological and economic advantages that favor its rapid adoption and integration into HCS [3]. 

The integration of AI in medical education enables the provision of learning and training services around the clock, breaking the bounds of traditional classroom schedules. It paves the way for more immersive, engaging, and personalized instructional methods, heralding a new era of educational experiences uniquely tailored to the needs of the individual student. Moreover, the current shortage of healthcare workers across various medical professions underscores the urgency of expanding training opportunities. Artificial intelligence in HCS may very well be able to help meet this rising demand for training without the proportional increase in faculty and instructional resources. Furthermore, the growing calls for enhanced training opportunities with better healthcare outcomes underscore the necessity of leveraging AI to maximize the efficiency and effectiveness of simulation training and assessment.

This review will begin with a concise summary of AI and its discipline-specific lexicon. Following this, we will delve into the current impact of AI on HCS-related training and education. However, one needs to caution the reader that any attempt to classify AI or separate it into distinct 'areas of influence,' where AI has demonstrated substantive progress, suffers almost instantly from two unavoidable sins, namely a dangerous trend toward oversimplification and a degree of artificial segregation that is not there. For example, one may wish to focus on the inroads made by AI in the field of surgical robotics, but how does one separate the role that AI also may have played in how residents acquired their expertise in the use of that particular robot? Or, even slightly more afield, how does one separate the role that AI may have played in a diagnostic support system that broadened the differential diagnosis to include a surgical one, which is now leading the patient into the operating room? We are not dealing with scattered, distinct areas of development but rather looking at an entire ecosystem that is being transformed under the influence of AI [4]. And, while, for the purposes of discussion, it helps to tease the subject matter apart into its components, one must also be able to see them as operating in concert. 

With these significant limitations in mind, the review will focus primarily on AI’s impact on simulation. It will begin by looking at 1) AI’s role in simulated patients, 2) AI-driven simulation targeting team skills (e.g., interprofessional education), 3) AI in procedural and task training (including surgery), 4) AI in simulated instruction, 5) AI in clinical curriculum alignment, and 6) the obstacles to achieving AI literacy. Throughout these subject areas, we aim to not only inform about AI’s applications but also inspire a rethinking of how AI can be harnessed to reshape the future of healthcare education.

A brief primer on the concepts and nomenclature of AI

It is essential to remember that AI differs from other forms of computer processing because it mimics human-like thinking and problem-solving. With natural language processing (NLP), AI can understand written and spoken languages without specific programming. It also excels in pattern recognition and predicting trends from large data sets. Finally, AI can automate tasks and processes without requiring additional manual intervention or programming. In short, AI is different from all computer platforms that preceded it for four reasons: 1) AI learns from the data it processes and can incorporate that learning to change its behavior or decision process; 2) AI's database(s) can be enormous and may, under some circumstance represent a substantial portion of knowledge available on the internet [5]; 3) AI can tackle numerous tasks without needing to be specifically programmed to undertake those assignments; and 4) under many circumstances, AI can write its own code to handle or complete a task. This combination of capabilities makes AI a game-changer. They lend AI different properties from anything we have encountered before in information technology.

Many individuals wonder why, in 2022, AI suddenly seemed to become a hot topic. First, the notion of AI per se is nothing new. It can be traced back to World War II when British mathematician and cryptologist Alan Turing developed pioneering notions about computers that could think like human beings. American scientist John McCarthy coined the term "artificial intelligence" in the mid-1950s. Several terms have been used either synonymously or in conjunction with AI. They include expressions such as 'machine intelligence,' 'cognitive computing,' 'autonomous computer systems,' and 'artificial general intelligence' (AGI). The latter most, i.e., AGI, is typically used to designate AI systems that possess human-like intelligence and perform most (if not all) the cognitive and intellectual tasks that a human being can. It is more theoretical than an actual concept, as most AI systems we currently work with would be termed 'narrow' or specialized. A final contributing father to the AI movement was mathematician John von Neumann, who gave the world stochastic computing theory, topology, and gaming theory. On his deathbed, von Neumann finished writing *The Computer and the Brain* in which he outlined the potential for an "arms race" between the human brain and AI technology. He coined the concept of "singularity" to describe the flexion point at which the pace of technological change could no longer be controlled by human beings.

A handful of other terms need delineation while addressing the application of AI. The first is the overarching concept of machine learning (ML) [6] which provides AI with the ability to automatically learn and improve from experience without being explicitly programmed. The development of computer programs that support ML means that AI can not only access databases but can also use them to learn for themselves [7]. Machine learning begins with reviewing the data itself (such as illustrative examples, direct experience, instructions, etc.) so AI can gain the capacity to look for patterns in the data and make better decisions in the future based on the examples or databases provided. Machine learning aims to allow computers to learn automatically without human intervention or assistance and adjust their actions accordingly. A handful of other terms also need delineation when discussing AI. The term reinforcement learning (RL) represents a subfield of ML where a computer learns to make assessments and decisions through interactions with the environment. A prime example would be an AI system learning to play chess. Here, the chessboard, the pieces, and the opposing player constitute the environment. When it starts, the computer system (an 'agent') randomly moves the chess pieces around. It progressively learns, for example, which moves are permitted, what may put a particular piece at risk, and the relative value of particular chess pieces. By playing and evaluating the outcome of each game, the agent progressively moves the pieces to achieve increasingly better outcomes. The AI platform learns the intricacies of playing chess using a random, trial-and-error methodology. An algorithm is a step-by-step instruction set by an agent to solve a specific problem or achieve a particular goal. A rubric, by contrast, is a set of guidelines or a scoring system that the agent uses to assess and evaluate the quality of a particular outcome or the performance of a designated task. In AI parlance, a model refers to a mathematical or computational representation of a particular process designed to simulate or predict a task or outcome in the real world. For example, models are used for image analysis, predictive analytics, or NLP. 

Artificial intelligence uses ML, which describes how a computer system can learn and improve from the data it processes without specific programming. Moreover, AI can feed itself more data and write its own code to further adapt its behavior and responses to that data. Deep learning is a special case of ML that involves deep neural networks that permit AI to process, analyze, and understand complex data sets. A neural network is a computational model inspired by the principles of neuronal connectivity in the human brain. A neural network comprises multiple layers of interconnected nodes to permit AI to carry out complex tasks such as pattern recognition or decisional support analysis [8]. The other important principle at work in AI is that it learns from large, complex data sets. If the way that data is collected or categorized is incomplete or biased in any way, then the processing that will be subsequently carried out by AI will also be flawed. 

One feature that dramatically altered the AI landscape in 2017 was the introduction of a new model or computer 'engine' called a transformer. Up until the introduction of transformer architecture, people were working on language, predictive analytics, or image processing, but each group tended to work in their silo. Transformers were introduced by Vaswani et al. in a landmark paper titled *Attention Is All You Need* [9]. It advanced the notion of a "self-attention mechanism" that permitted the AI model to weigh the importance of different words or values in a sequence. In essence, it meant that any data could be converted into symbolic language where AI would attempt to predict what item (word, pixel, etc.) would most likely appear next in a given series. With the introduction of transformers, tasks could be broken down into a common representational language, and all the various disciplines and functions of advanced computing were able to communicate with each other. This was a breakthrough because it gave computer systems devoted to AI the ability to capture contextual information, model long-range dependencies (semantic relationships in a sequence), and perform these operations in parallel [10]. Transformers brought us what we know as the generative large language multimodal models (GLLMMs, pronounced 'Gollum,' like the character from *The Lord of the Rings*) that can process and generate content across a wide range of modalities. The GLLMMs can also generate creative content such as artwork, music, poetry, etc. [11] Because they are also typically pre-trained on very large data sets, GLLMMs can be quickly applied to other fields or tasks without requiring training for the agent from scratch, so they are much more quickly deployable to specific tasks. The kinds of tasks to which GLLMMs can be applied include pre-training in content creation (such as text, audio tracks, images, or videos) that become part of a larger undertaking such as designing art, composing music, or telling a story. Another example would be language translation where GLLMMs can convert text from one language to another with greater accuracy because they have also been primed by gaining earlier access to cultural nuances and context [12]. An example where GLLMMs are applied in the field of medicine would be to analyze the results of medical imaging more accurately (such as X-ray, MRI scans, or ultrasound) by pre-training with access to additional databases that relate to the relevant histology and pathology implicated in the imaging results [13].

## Review

The current status of medical simulation 

Artificial intelligence plays a crucial role in enhancing training, planning, and treatment strategies in what are already well-established areas of HCS [14]. The first is one-on-one training honing bedside skills and decision-making with individual patients. The second broad category is scenario-based training which calls for group/team responses. These may be led by an individual, but that individual is responsible for the conduct of the teams for a particular scenario. Team responsiveness and effectiveness are measures of how well interprofessional education (IPE) skillsets are applied to ensure the appropriate behavior of team members is enlisted. The third category is task or procedural training (PT). To the extent that practice with a task trainer engenders familiarity, and sufficient handling of instruments imparts practiced expertise to the healthcare professional (HCP), simulation is commonly used to assess surgical or technical proficiency. In conclusion, simulation can be broken down into two broad categories, namely individual patient care and task or PT. These simulations call for additional participation of personnel, thereby resulting in professional team skills acquisition as well (Table 1). Artificial intelligence-driven instruction in simulation can be applied in several different formats, such as virtual patients (VPs), virtual instructors (VIs,) instructional games, and so-called intelligent instructional tutoring systems (IITS). In older educational lexicons, IITS was also known in its earliest iterations as computerized programmed learning, and later, as adaptive learning systems [15,16].

**Table 1:** Types of simulation training IPE: Interprofessional education

Case or patient scenario				Task or procedural training	
	Standardized patient	Computerized manikin	Virtual patient	Simulated task trainer	Virtual task or surgical trainer
Single	Yes	Yes	Yes	Yes	Yes
Pair	No	Yes	Yes	No	Yes
Team (IPE)	No	Yes	Yes	No	Yes

AI for clinical skills and patient care

AI and Standardized Patients (SPs)

Standardized patients currently form the backbone and gold standard of most preclinical teaching programs, where interviewing and clinical examination skills assessment are part of the preclinical curriculum. Standardized patients are individuals who have undergone preparation and coaching under professional trainers to be able to role-play a specific subset of patient characters who are suffering from a series of ailment-related symptoms and manifestations. Initial studies [17] in the early 1990s evaluated whether SPs presenting as new patients in an ambulatory clinic could be differentiated from real, actual patients. In the majority of cases (>80% of the time), treating physicians could not detect any differences. Williams [18] has also suggested that the more trained and experienced the SP was, the more likely it would be that they would escape detection. As for the accuracy of the 'case portrayal,' SPs were deemed to have performed the assigned malady well in 90% of the cases, but variability in the portrayal is a source of constant concern. This variability is of deeper concern when it comes to issues of gender and ethnicity biases in the SPs’ evaluation of clinical skills [19]. There were also glaring incongruities between first-year residents’ self-evaluation of their empathy and their scores on the Jefferson Scale of Empathy and how the SPs assessed their empathetic skills. 

Standardized patients are also trained to provide constructive feedback about student bedside techniques and behaviors. While there is little doubt that having SPs role-play patients produces more consistent and cost-effective results than having faculty perform this task, the costs for SPs for a single medical school, especially those that have incorporated SPs into the Objective Clinical Skills Examination (OSCE), can run well over a thousand dollars per student [20] and represent annual institutional costs that can be well into six figures [21]. Not only is the provision of SPs for OSCE an expensive proposition but there is also a wide range of disagreements over what checklist must be followed [22]. The best results are still derived by a panel of three to five physicians who are subject matter experts (SMEs), but panels that large present logistical problems when it comes to arranging for an entire class of medical or nursing school students at one time. It also requires substantial funds to be set aside for SP personnel as well as for faculty time away from clinical work. Standardized patients, however, continue to have an important place in the first years of medical school, and there is a reasonable correlation between how students were ranked on their OSCEs as students and how they were seen later to perform as trainees by their respective residency directors [23]. 

AI and Virtual Patients

One of the most important transformations that AI is bringing to HCS is the plethora of new AI-driven virtual patients (VPs) [24]. Virtual patients have begun to manifest themselves in the healthcare curriculum in different guises. The most common setting for a VP is an interactive, case-based scenario. The format employs either 2D screen-based avatars or 3D AI-driven VP avatars [25] that can be visited either in an immersive environment or even as a holographic representation [26]. Virtual patients are becoming an increasingly vital component of simulated training across all disciplines and levels of experience. 

There are many advantages to employing VPs [27]. These include reduced initial equipment outlays, easy accessibility (students have 24/7 access to patients and cases), standardization in presentation and evaluation, and decreased labor costs for both educators supervising clinical settings and the staff hired as SPs. In addition, VPs offer the built-in reassurance of scalability (both concerning the number of students, progressive difficulty, increasing expertise, and specialization). These advantages grow with the addition of AI because it enhances interactivity and engagement while also providing almost immediate feedback and coaching [28]. While some people wonder if a mannikin is not a VP, simulation mannikins are usually not included in a discussion of VPs, although that position may have to change shortly with the advent of increasingly animatronic mannikins. 

Artificial intelligence allows health educators to create an almost infinite number of case scenarios and patients with specific objectives and assessments built into them [29]. In the past, it was difficult to design AI-driven scenarios to include parameters such as cultural inclusivity, diversity, measures of empathy, and mutual respect [30]. The AI-driven VPs can be used to help students probe for explicit or implicit biases [31] that often find expression in differences in analgesic administration, empathetic communication, and even in answering questions from family and patients. Artificial intelligence can be used to create VPs that specifically respond and provide rapid feedback about a medical student’s expression of empathy during an interview. Furthermore, such training translates to significantly higher expressions of empathy when working with SPs [32]. Finally, AI permits the creation of 3D-printed patient-specific models derived from the patient's imaging studies. Such models can assist surgeons in preoperative planning. The AI-derived rapid prototyping of patient-specific anatomy for lumbar spine surgery has been used as part of the procedural consenting process to make the operation easier for patients to understand and assist trainees by permitting them to view image-based anatomy before the actual surgery [33]. 

With the funding support of the National Board of Medical Examiners, Ohio State University created a VP that employed a gaming engine to create 3D avatars on demand in conjunction with a conversational engine so that the patient characters could be interviewed by medical students to create a patient encounter [34]. Relative to the case generated by AI, the conversant responses were accurate between 79% and 86% of the time. A simulation-based research group in Sweden has also developed VPs that permit enriching the avatar with differences in ethnicity, culture, or socioeconomic status [35]. This offered students an opportunity to repeatedly visit issues of diversity, inclusivity, and limited access to healthcare at a time when the entire European Union was facing increasing pressure from influxes of refugees. 

The Institute for Creative Technologies (ICT) at the University of Southern California uses NLP to create a VP designed to enhance the interviewing skills of both doctors and therapists. It also focused on ensuring the interviewers were reinforced by including verbal acknowledgments, relying on open-ended questions, using non-judgmental questioning, and employing empathetic reinforcement with body language to engender a greater sense of trust in their patients [36]. In a kind of 'virtual emotional reverse engineering,' ICT also created a virtual interviewer capable of recognizing verbal cues, facial changes, and body language to enhance support from the human patients that it interviewed [37].

More than the simple growth vectors in the markets for AI and VPs, there are decided camps among clinical instructors in medical school beginning to form around their experiences employing SPs versus VPs. In one study, the teaching faculty felt VPs offered more opportunities for safe, repetitive training, and they seemed to engender more self-directed learning and more instances in which to enhance clinical reasoning. Standardized patients, on the other hand, seemed to excel in teaching team-based communication skills. While VPs offered a major advantage in terms of saving labor, cost, and time, the authors of the study suggested that the best outcome would lie with faculty learning to selectively exploit the advantages of each modality to achieve their objectives as clinical instructors [38]. 

Often, studies have shown how hard it is to make valid, direct comparisons between AI-driven VPs and human SPS. For example, in one study [39] that used a randomized crossover design, medical students were taught about the delivery of high-value care to both kinds of patients. The investigators found that working with VPs seemed to preferentially enhance students’ skills for interviewing and delivering treatment. By contrast, the student’s success working with human SPs correlated more closely with the student’s basic fund of knowledge in science and clinical areas, as it might be called upon for taking Steps 1 and 2, respectively, of the United States Medical Licensing Examination (USMLE) [40]. In short, these results suggested that a human performance system (HPS) called for a greater draw of basic and clinical facts, while VPs might be better suited to practicing bedside communication skills. In another study, looking at primary care across seven cities in China, the learners were confronted with a patient to be evaluated: either an unannounced VP or SP in the clinic, both representing the same clinical case. Despite a relatively large sample size, variables such as location and case condition influenced the outcome of the learner’s performance, and there appeared to be little correlation between the VP and SP encounters. Certainly, with the new capabilities to use AI to carry out detailed evaluation and assessment, there will need to be more thorough research to sort out the respective advantages of VPs and SPs.

AI and Virtual Instructors

The use of AI does not stop with VPs. Artificial intelligence is just as comfortable working 'the other side of the aisle,' meaning that AI could just as easily inhabit the physician in a bedside or clinic interaction as the patient [41]. Several studies have evaluated the impact of VIs [42]. Being able to switch back and forth at will between VPs and VIs took on a new urgency during the isolation imposed on students and physicians during the COVID-19 crisis. For example, in one study, 64 nursing students were randomized to receive their mandatory sepsis team training under the tutelage of a VI or a real physician. Both groups underwent baseline testing before being enrolled in the sepsis training. After the training was complete, both the VI group and human instructor (HI) groups were assessed on their fund of knowledge, the efficacy they demonstrated in team communication, and their sepsis care in a simulated case. Both groups demonstrated significant improvements in their post-training testing, and there was no difference in sepsis care performance between the VI and HI groups. However, the VI group demonstrated a significant improvement in their fund of knowledge compared to their HI counterparts. In this particular setting of sepsis team training, providing a human physician instructor seemed to offer no advantage. Similarly, in a larger study carried out at the Singapore National University during the COVID-19 pandemic, over 415 medical and nursing students underwent instructional sessions using a remote virtual tele-simulation platform to enhance interprofessional sepsis team communication skills, and both groups of students demonstrated significant improvement in post-instruction test scores and their team communication skills [43], again reinforcing the finding that VI were not inherently inferior or less successful in achieving measurable educational objectives than human instructors [44]. Again, with continued research, we would hope to predict areas and methodologies where there may be parity between VIs and HIs, as well as uncover areas where each of the respective teachers might excel.

AI and Intelligent Instructional Tutoring Systems 

Instruction by AI is much more widespread than by VIs. Artificial intelligence chatbots are ubiquitous on today’s university campuses. They are no farther away than our cell phones, and every one of them represents a customized, personalized mentoring system. Students query them without the least hesitation (which may be imprudent in some cases), but it is still a testament to how naturally young adults gravitate toward this unhindered access to learning. Chatbots are the teaching technology of the new age. Artificial intelligence algorithms and ML rubrics of ML have established a new standard for these chatbot-based mentorships. They are universally available, have unimpeded 24/7 access, can provide customized coaching, are scalable to increasing numbers of users, and can adjust as the student’s mastery grows [45]. Many in education have noted that students seem more willing to adopt a more collegial or collaborative attitude toward their chatbots than, say, with earlier interactions with simple search engines [46]. 

Mentoring has played a key role in professional education and training. It ensures progressive exposure to technique and professional demeanor, placing increased demands as the trainee’s proficiency increases with the eventual goal of mastery. Modern NLP algorithms allow learners to literally converse with chatbots, and many mechanisms permit the user to personalize their bot’s settings to customize their mentorship experience by favoring user profiles, contextually relevant learning algorithms, and adaptive learning paths [47]. These settings should help the user ensure the AI mentor focuses on appealing to the user’s needs, preferences, and learning objectives while remaining responsive and engaging [48]. The addition of AI mentors is even more notable as the availability and participation in online instruction continue to increase. The use of chatbots makes participation in the online formats more attractive to students and enhances their sense of self-satisfaction [49], while also providing an excellent way to profile how students are progressing with mastering the material online [50].

AI and Chatbots

It has been suggested that the thought leaders in today’s world of medical education may have if anything, grossly underestimated the extent to which chatbots have penetrated the lives and learning styles of our medical and nursing students [51]. A.I.-driven chatbots can accomplish a host of educational housekeeping chores: everything from turning lecture notes into flashcards for review, reminding students of useful and pertinent acronyms or mnemonics, or isolating facts and figures likely to be on qualifying examinations like the USMLE Step 1 and Step 2 [52]. Chatbots also serve as instant sources of internet-derived information which help flesh out workups and double-check differential diagnoses. But it can also provide a ready-made list of pre-op orders, issue cautions about drug interactions, emphasize dose adjustments, or even accelerate the delivery of test results or radiographic findings directly to the bedside [53]. This next generation of HCPs will have a different sense of intimacy with medical facts and data as well as an urgent impatience to see data woven into coherence before their eyes at the speed of light.

AI and interprofessional education

AI and Gamification

The process of gamification refers to the application of the elements of game design into what were or are traditionally non-game contexts in the hope that gamification might help improve learner engagement, motivation, and participation [54]. The overarching principle of gamification is that the use of external rewards embedded in the game experience (e.g., new levels of higher access in the game, additional points, powers, etc.) serves to eventually support a higher level of intrinsic motivation. It has been said that the success of games revolves around their ability to engender 'the 3 Fs', namely, fun, friends, and feedback [55].

Employing AI in gamification allows for the game to become more personalized and rapidly adaptive to the needs of the individual [56]. Artificial intelligence analyzes the user’s data in real-time to tailor challenges, rewards, and feedback to ensure the experience is engaging and effective for individual users. Artificial intelligence-driven analytics can predict user preferences and adapt gamification strategies in real-time, enhancing learning outcomes, user satisfaction, and the overall effectiveness of gamified applications.

Artificial intelligence-driven games have been developed across the healthcare sector [57] to address such diverse issues as motivating and charting progress in the first-of-its-kind FDA-approved game (EndavorRx, Akili Interactive, Boston, MA, USA) to enhance attention in youth suffering from attention deficit hyperactivity disorder (ADHD) [58]. Another game, Re-Mission (Hopelab Foundation, San Francisco, CA, USA), helped children with cancer better track symptoms that arise from their disease versus those relating to the toxicity of treatment and sought to improve the compliance of younger children [59]. Artificial intelligence-enhanced games have also been used in rehabilitation [60], specifically in the testing and reinforcing of recovery in motion tracking after a stroke [61], as well as plotting and following cognitive impairments [62] after a stroke.

Concerning AI-driven games that support medical simulation and training, one specific game (Fanta Training®, Brain Refresh Lab, Milano, LM, ITA) in pediatric obstetrics and gynecology takes trainees through a variety of scenarios where obstetrical anesthesia may be required [63]. While playing the game, players are also peppered with quiz-type questions where they compete against other players for points. Open-team competitions that pit one group of trainees from one medical program against another have flourished, including Sim Wars, hosted by the Society for Simulation in Healthcare (SSH), where teams are provided with a clinical scenario and must respond to it and treat the patient against the clock. A similar competition was developed by Stanford to encourage skill acquisition by residents in point-of-care ultrasound [64]. The potential applications of AI-enhanced gamification are almost limitless: it has been applied to teach basic dermatologic diagnoses [65], on-boarding new nursing personnel [66], and the application of resuscitative techniques in patients who have recently undergone cardiothoracic surgical procedures [67]. However, the concept of scalable AI-enhanced gamification is a methodology that is still so new as to have largely gone untested [68] with respect to its efficacy in transmitting a fund of knowledge, enhancing the acquisition of technical skills, or maintaining proficiency once a trainee has attained it. 

One would be hard-pressed to describe the notion of building a digital twin for a given app or simulated therapy as a 'game.' However, the circumstances surrounding creating digital twins and their environments are reminiscent of what developers do in the process of mapping out and building a game. The concept of creating digital twins emerged from certain industrial manufacturing and engineering disciplines, where it was used to create simulations of physical objects or systems [69]. It became commonplace, and better computing capabilities allowed engineers to employ digital twin testing to evaluate materials, such as looking for metal fatigue in airplane parts. The use of digital twins in the study of clinical causality training refers to the creation of highly detailed and dynamic digital representations of patients that are used for simulating and analyzing the effects of various medical interventions, treatments, or drugs. For example, the creation of a digital twin for a cancer patient to evaluate the impact of multi-modality chemo and radiation therapy and to measure the effect on the patient's overall health versus, perhaps, causing an increased therapeutic response in the tumor bed [70].

AI and Teamwork Assessment

Artificial intelligence has created opportunities for IPE and practice, where scenarios (both clinical and non-clinical) are developed to challenge interdisciplinary teams to solve problems, overcome obstacles together, and decide on a course of action. These scenarios stress teamwork and leadership skills. Artificial intelligence can be applied in IPE exercises to help evaluate five areas of team effectiveness as developed by the Agency for Healthcare Research and Quality (AHRQ, a part of the US Department of Health and Human Services) [71] that include measures of communication, member assignments, leadership, mutual support, and situational awareness. Artificial intelligence has also been more recently configured as a virtual facilitator for IPE training with the objective of reinforcing the AHRQ objectives [72].

*Bedside Communication Skills With VIs * 

In terms of the perioperative environment, immersive virtual reality (IVR) has allowed students and trainees to interact within specialized environments, such as the post-operative acute care unit (PACU), the intensive care unit (ICU), the ambulatory clinical setting, and the emergency department (ED). Immersive virtual reality has also been applied with an AI agent to provide exposure to and training for the human skill sets that come into play outside the operating room (OR). Such platforms (e.g., Virti, Bristol, UK) allow HCPs to converse with the patient, discuss operative options, or carry on a discussion with family and patient at the bedside. Artificial intelligence and NLP allow the platforms to have realistic conversations. In addition, AI monitors the progress of the HCP concerning empathy, communication skills, and situational awareness and can adapt the scenario on the fly to the trainee's level of proficiency [73].

AI and Simulated Pediatric Referrals

Increasingly, there has been an emphasis in healthcare education on team dynamics and interprofessional communication skills. Enhanced communication is amenable to being taught and practiced like any other skill set. Cincinnati Children's Hospital developed 21 AI-generated scenarios using the IBM Watson AI platform (IBM Watson Group, New York, USA). It provided a conversational exchange in which pediatric residents in their ICU rotations were called upon to communicate in real-time with a simulated primary care physician (PCP) about a fictitious pediatric patient who was being transferred to the ICU. The pediatric residents were instructed to include and discuss four specific areas: 1) the nature of the principal problem; 2) the plan of care for the patient; 3) the anticipated discharge date for the patient; and 4) to solicit any additional or ancillary information from the PCP that might impact the care of the child while they were in the ICU. The AI engine also evaluated the residents on how successfully they carried out the objectives of the conversation and provided each resident with individualized feedback (pre-verified by attending teaching staff). The AI group was compared to a control group that had received the usual didactic training on conversational objectives with PCPs during their internship year. Simulated exchanges were considered equal to or more effective than reading instructional materials, online didactics, or real-time observation. Only live simulated encounters where the PCP was role-played in person were ranked as superior to the AI-simulated experience [74].

AI for procedural, task, and surgical training

AI and Surgical Simulation

The context of most traditional surgical simulation training is one in which the trainee is usually assured a certain level of guaranteed supervision from their supervisor while also putting the faculty member at a significant disadvantage, as one-on-one mentoring during designated sessions will limit how much time each student gets and how equitably that attention is shared among participating learners. The McGill Neurosurgical Simulation and Artificial Intelligence Learning Centre (MNSAILC) in Montreal, Canada, has a long history of using its expertise in neurosurgical simulation to design studies that permit the more general user in the field to benefit from the findings. For example, the research staff at MNSALIC carried out studies to see how an AI-driven operative assistant would compare to its human counterpart [75]. The medical students were randomized to a series of five operative sessions where they would receive procedural training from an experienced surgical instructor. By contrast, the other group of students would be directed and supervised by an AI-driven operative assistant that employed assessment algorithms that allowed close monitoring of students’ progress before, during, and after each training session. The performance of students from both groups was tested on completion of the training. Expertise scores were significantly higher (p <0.01) for the group that received its instruction from a virtual operative assistant (VOA). Operative Structures Assessment of Technical Skills (OSATS) scores were not significantly different between groups, but OSATS sub-scores for instrument handling were higher in the VOA group. No differences were seen in any of the emotional self-satisfaction questionnaires. The researchers concluded that students who were taught by AI-driven virtual assistants “demonstrated superior performance outcomes and skill transfer" [76]. This would seem to bode well for not only the efficacy of turning over procedural instruction to AI virtual assistants but also suggest that substantive cost savings could be achieved in the long run. This opinion is shared by other experts who have compared the institutional advantages of AI-driven instruction compared to traditional surgical simulation expenses [77,78]. 

AI and Immersive Virtual Surgical Training

Currently, several platforms can employ AI to provide IVR for an immersive surgical training experience that can be custom-tailored to the individual needs of the trainees. Osso VR (San Francisco, CA, USA) utilizes an extensive library of virtual procedures, allowing surgical trainees to assemble individual portfolios of the required operations and procedures. In addition, the level of expertise required by the individual trainee can be carefully adjusted by tracking their earlier progress on preceding procedures and modifying the difficulty of the surgical task according to each trainee's learning curve [79,80]. A small randomized clinical trial of reverse shoulder arthroplasty (RSA) compared senior orthopedic residents trained with an IVR trainer to a similarly senior control group that received traditional training based on surgical videos. At the end of the training period, both groups were brought to a cadaveric facility to carry out the RSA on a specimen from a fresh, frozen cadaver. The residents were evaluated by pre-trained, masked evaluators blinded to the randomized group assignment. They scored the residents on implant parameters, such as implant rotation and overall task completion time. Residents trained on the IVR platform completed their procedures at significantly faster rates and performed statistically better on the cadaveric procedure assessment (p <0.01). The control group also committed 50% more critical errors than the cohort that trained with the AI-IVR program [81].

AI and Surgical Mentoring 

There is a dramatic upswing in AI-driven assessments of surgical procedural proficiency. Although the notion is still in its infancy with AI-driven surgical simulation, many educators are increasingly skeptical of allowing AI to perform assessments without having better access to transparent decision algorithms [82]. Researchers at MNSALIC assessed the validity of an AI-driven surgical assistant to provide residents in training with automated feedback on their technical performance of an IVR-based subpial brain tumor resection. The performance measures were compared to the metrics obtained by a panel of experts performing the same simulated procedure. The AI-driven operative assistant successfully differentiated skilled versus novice participants using four metrics with accuracy, specificity, and sensitivity of 92%, 82%, and 100%, respectively. A two-step feedback system was developed to provide participants with an immediate visual representation of their standing relative to expert proficiency performance benchmarks. The researchers felt that transparent algorithms used for assessment allowed educators to categorize the relevant psychomotor components contributing to technical proficiency carefully. It was felt that such an approach was germane to surgical training, where hands-on experiential learning significantly contributes to overall competence [83].

The MNSALIC randomized medical students into one of multiple arms for evaluation of the resection of a simulated tumor. The first group received AI audiovisual instruction along with metric-based feedback (VOA group), the second group received synchronous verbal scripted debriefing and instructions from a remote expert (instructor group), and the third was a control group that received no feedback. The study focused on two sets of measures. The first was an AI-generated expertise score. The second was an objective assessment of expertise carried out by blinded observers. There was also a self-report on emotional and cognitive load before, during, and after the intervention. The VOA group demonstrated superior performance outcomes and skill transfer. There was no difference between the objective expertise ratings from the blinded observers or in the self-reporting of cognitive and emotional workload between the VOA and instructor [76].

AI and the Future of Surgical Mentoring

The MNSALIC extended some of their research efforts to use AI to evaluate trainees’ neurosurgical skills using a virtual hemilaminectomy [84]. The study employed an algorithm designed to accurately assign a level of expertise to each participant using leave-one-out cross-validation (LOOCV). The LOOCV is a method applied in the evaluation of a model used in ML. In a given set of data points used for validation, each data point is used as the validation set, while all the remaining data points are used to train the model. It is especially useful when applying the model to training data [85]. Not only can such standards be applied to an infinite catalog of procedures, but ML learning can apply a similar methodology to evaluate technical surgical skill sets derived from simply analyzing videotaped recordings of actual procedures [86]. Naturally, such AI models could then be used for determining surgical proficiency but also for instructing robots on how to carry out competent surgical maneuvers. For example, the Smart Tissue Autonomous Robot (STAR), developed by Johns Hopkins University (Baltimore, MD, USA), is an autonomous robot capable of performing intestinal anastomosis with minimal human interference. The robot carries out an analysis of the surgical field and then can choose from a host of autonomous surgical strategies before selecting which surgical technique it will employ. This machine “outperforms expert surgeons’ manual technique and RAS (robot-assisted surgery) technique in terms of consistency and accuracy" [87]. The evolution of modeling and assessing surgical technical skills sets the stage for eventually being able to produce AI-driven, skilled surgical robots capable of assisting surgeons in the operating room or autonomous surgical robots suitable for battlefield or space missions.

AI and Curriculum

We have seen how AI can shape matter, facts, and even teams of practitioners. Artificial intelligence will be ubiquitous in the medical school classroom, at the patient’s bedside, in ambulatory clinics, and throughout simulation facilities for assessment. However, to date, little formal instruction is being built into current medical school curricula or postgraduate training programs to ensure that our students and trainees acquire a modicum of AI literacy and are fluent with the most frequently encountered AI apps in their own specialty [88]. A second alternative method of study for students to gain AI literacy is to ensure that both didactic material and practical lab sessions about AI are included as part of a structured, longitudinal curriculum in medical or nursing schools [89,90]. Furthermore, AI is adept at helping to create easily administered pre- and post-exposure (or training) assessments [91]. The effectiveness of the curriculum at translating curricular objectives can easily be correlated with the students’ happiness in experiencing the course, and both can be linked to measures of students’ success as determined by serial assessment. The injection of AI into the course in all these manners of processing, satisfaction, and evaluation can make the reshaping of curricular material rapidly responsive to a short feedback loop while delivering to the end users a customized assessment that is unique to them [92]. By its very nature, AI deemphasizes rote learning while underscoring increased active participation and interdisciplinary team engagement [93]. 

Reshaping curriculum with the help of AI requires a balancing act. On the one hand, one must weigh the adept and facile manner in which AI can monitor the ability of the curriculum to deliver educational value and return in real-time, and on the other hand, one needs to also ensure that AI is allowed to deliver customized, responsive feedback that ensures learners are the beneficiaries of a training system that builds on their personal strengths while addressing their weaknesses [94]. The application of AI to help the curriculum evolve demands an honest and ethical appraisal of the best use of education resources and instructor/facilitator time so that the students can avail themselves of the ascendant emphasis on feedback and counseling [95]. A meta-analysis [96] of 257 papers describing the effects of AI-driven decision support systems in public health protocols at four different institutions showed that when students used these systems, there was (a) enhanced adherence to evidence-based guidelines; (b) improved, sustained supervision by faculty; and (c) diminished medication errors consistently demonstrated. There were also trends in diminished utilization of care and improved preventive health measures being put in place. This analysis could not draw consistent conclusions about cost savings. Overall, there is a general consensus that AI technology has a net positive impact on both the quality of instruction provided by teachers and on the learning experience and outcomes of students [97].

Artificial intelligence can also produce unique training solutions that are hybrid blends of ML built around past team responses in combination with a diagnostic decision support system (DDSS) to determine when and how a team is called into action. In the studies by Dillon et al. and Wheeler et al., prior experiences with a rapid response team (RRT) resulted in typical practice sessions built around scenarios drawn from advanced cardiac life support (ACLS) training [98,99]. Artificial intelligence has made several inroads in improving outcomes for training and deploying an RRT, but one of the key components is using ML to effectively monitor vital signs, electrocardiogram (ECG) tracings, and other critical care functions around the hospital. Such an AI-driven monitoring system increases the likelihood of a pre-emptive notification of RRT personnel while also dramatically reducing the mean alarm count per day to which the team must respond. Perhaps most significantly, the AI monitoring meant that team responses could be selective rather than always activating the entire team to every alarm. The AI-based warning system proved to be 259% more sensitive in identifying potentially unstable patients than the hospital protocols that had been previously in place [100].

Finally, AI will be used to teach learners traditional skills, but with a greater emphasis on individual mastery and assessment. For example, medical students need to acquire the ability to recognize pelvic fractures. To accomplish this, an AI-enhanced instructional platform was created to assist students in learning how to diagnose hip fractures from standard pelvic X-ray films. Through repetitive practice, the AI instructor highlighted salient features that should be looked for and identified in the films. Medical students were randomized into either an AI-enhanced instructional group or a control group that received conventional didactic sessions and materials. While the pre-learning tests were equal between both groups, the AI group demonstrated significantly more improvement in its post-test evaluations [101]. Artificial intelligence instructional programs will need to be developed and tested for their effectiveness in supporting the needs of the curriculum.

AI and Diagnostic Decision Support Systems

Perhaps there is no more daunting concern than grasping how HCPs will be taught to incorporate AI into the very foundation of their clinical reasoning. There is no doubt that HCPs will need help to better understand when and how to bring AI to bear on issues of patient care. One of the most important avenues available will be to provide simulated clinical scenarios where HCPs can assess for themselves the impact of using AI as an adjunct to clinical decision-making. One such study [102] was undertaken to evaluate if medical students enhance their diagnostic and decision-making skills by consulting an AI platform named Isabel (Isabel Healthcare, Ann Arbor, MI, USA). Students were assessed as they evaluated a series of specific patient simulation scenarios. After each case, students were asked to present their diagnostic hypotheses before consultation with AI (pre-Isabel) and then re-present their assessment after consultation with the AI platform (post-Isabel). The quality of the pre-Isabel differential diagnosis was compared to that which was presented after AI consultation (post-Isabel) to determine the impact of a DDSS on each student's clinical reasoning. A follow-up survey and focus group identified student perceptions about using a DDSS in educational settings. Paired t-tests demonstrated that diagnostic accuracy significantly improved after using the DDSS (p <0.05). Students found the software relatively simple to use and felt it helped them reflect on diagnostic options they had not considered initially. They valued the opportunity to train with the AI platform and be able to apply the DDSS in a simulated training situation. This kind of effort to build up confidence in new AI habits will thrust scenario-based simulation training into a brand-new role of demonstrating and validating the use of DDSS in routine patient care. 

AI and issues of responsibility and accountability

AI and Unexpected Consequences

There are a host of ethical and legal issues surrounding the development of AI and its applications to patient care [103]. Numerous authors and subject matter experts have raised the question of how medical schools (and other health profession schools) will address the looming issue of AI literacy [104]. Many educators have raised the theoretical question of how dependent learners should be on AI platforms for diagnosis and treatment. What happens when a physician relies too much on a fallible AI program and makes a critical error? Finally, some researchers have suggested that incorporating AI into the bosom of the medical culture may have unintended consequences. One study of simulated tumor resection allowed a group of neurosurgical residents to be trained in conjunction with an AI-enhanced curriculum that was meant to reinforce specific AI-selected technical competencies. True to form, the trainees in this AI-guided arm did much better on the four competencies than the trainees in the control that had trained without any highlighting of benchmarks. However, a global evaluation also indicated that other metrics showed significant decrements in operative performance among members of the AI group, including deficiencies in the amount of tumor resected [105]. These results should remind us that not everything associated with AI is a de facto positive outcome. Scientists and educators must remain sufficiently skeptical, wary of confirmation biases, and prepared to perform the requisite studies to ensure ill and positive effects are pursued with equal zeal and objectivity.

AI and Hallucinations

Artificial intelligence bots can be plagued with malfunctions known as hallucinations. The term hallucination describes an occurrence where an AI system generates an anomalous answer or result that is disconnected or unrelated to the expected operation of the bot. The result may appear immediately to be false. Exactly how hallucinations are generated is largely hypothetical. It may be the result of a misinterpretation of the data or an exaggerated extraction from the data. Other causes of hallucinations include contextual error, overly generalized conclusions, and errant feedback loops [106]. But these hallucinations can be substantive: seeing nonexistent objects in images, making inaccurate and false statements, or drawing wild, speculative conclusions. The unexpected appearance of these kinds of aberrant behaviors is unsettling in the context of medical applications of AI [107].

AI and Its Ethical and Legal Ramifications

While it is hoped that AI will increase the safety and availability of healthcare, it is paradoxically recognized that it poses significant challenges and threats to the ethical and legal guardrails surrounding the practice of healthcare and the protection of students within its educational and training programs. In part, this threat stems from the staggering amount of data that fuels the engine of ML. This creates an uneasy equilibrium between the desire to have access to vast databases, on the one hand, and the constant requirement to ensure that records have been rendered adequately anonymized and sanitized, on the other. In a position paper on AI and data privacy, experts drawn from across Europe concluded, “The development and implementation of AI for healthcare comes with trade-offs: striving for all-embracing data privacy has proven incompatible with the desire to realize the full potential of AI for medical problem-solving purposes” [108]. These threats are all the more urgent because the adoption of AI is far outpacing early efforts to create robust ethical and legal guidelines to govern its use. In addition, the scale of AI's applicability is so broad that it is unlikely one set of principles can cover everything. A second factor arises when we consider that bias can lurk in every database. These biases may over-represent the needs and issues of one segment of society while underperforming for and discriminating against another [109]. While consent and privacy are two of the most important principles governing the ethical delivery of healthcare and the conduct of its practitioners, AI operates in such a way as to challenge those tenets in two fundamental ways. The first is that ML operates largely on the premise that it can make decisions and exercise its own assessment without being specially programmed for any particular or exclusive application. The second factor stems from the fact that human beings can only penetrate so far and so deeply into the mechanisms whereby AI processes data [110]. Indeed, a black box truly lies at the heart of AI, and there have already been instances where AI bots have written their machine code that was indecipherable to their human creators [111]. Another ethical consideration is the issue of informed consent: does a doctor have the right to evaluate a patient’s data by employing AI without first informing the patient [112]? There has also been concern expressed about how AI might affect the bond of trust between healthcare providers and their students, trainees, and students who may see over-reliance on technology as a cause for losing confidence [113].

AI and Proximate Causality

The notion of using an AI platform that processes, analyzes, evaluates, and, in some cases, acts on the very support systems that affect our patients begs the issue of who (or what) is responsible for the consequences of actions impacted by AI. Obviously, the ultimate decision to pursue a particular treatment plan rests with the physician and not ML. The lack of transparency in the bot’s algorithms means part of its decision-making processes is a true 'black box' [114]. We should remind ourselves that bots don't go to jail; humans do. The decisions about the well-being of human beings rest with human beings. Legal scholars and experts in tort law have voiced concern that while AI may, in fact, streamline many liability concerns, it may also produce a new array of experts to evaluate the impact of integrative AI in the care of an individual patient [115]. Many experts believe that mistakes are inevitable and that, while the use of AI may help reduce errors, it may also permit large increases in volume and, thus, greater denominators for assessing risks. This is particularly important in fields like radiology and pathology, where computerized image evaluation with AI can increase the throughput speed for studies by tenfold or more [116]. 

AI and Inadvertent Data Exposure

The possibility of causing harm with AI is significant when considering that GLLMMs, by their very nature, can access extensive and disparate data sources, making protecting an individual far more worrisome because there are many avenues by which information can be obtained and routes whereby it may be exchanged, distributed, or stored. The sources for the leakage of identifiable information through the handling of databases by AI are myriad [117]. The volume and sources of data can be difficult to track. In addition, the thoroughness of procedures used to sanitize healthcare records can be quite variable, if not undependable [118]. Since the stored data may be part of a broader learning process for training the bot, there would be an opportunity for the AI agent to reflect its knowledge of the database in its responses. All data that is sent to open AI sources should be treated as if it could be potentially exposed to the internet.

There is an urgent need to develop sufficiently solid and broad ethical and legal guidelines or guardrails to facilitate the application of AI technologies while still protecting the privacy of patients under the provisions of the Health Insurance Portability and Accountability Act (HIPPA) and safeguarding the rights of students and trainees under the standards of the Family Educational Rights and Privacy Act (FERPA) [119].

AI literacy and the future

There is a majority consensus among faculty and medical students that they wish to become fluent in AI, especially regarding education and patient care [120]. Simulation takes on added importance as it offers students and faculty opportunities to appreciate the principles behind AI and try its many applications on simulated patients. 

The application of AI to medical simulation will bring several direct advantages to the simulation itself. Artificial intelligence will permit more personalized and adaptive learning and offer much greater opportunities to tailor task training to the curricular needs and the individual student's experience. Future medical simulations will leverage AI to create hyper-personalized learning experiences, adapting to a trainee's performance, learning style, and career focus in real-time [121]. Artificial intelligence will enable simulations to reach new levels of realism and interactivity, with virtual patients exhibiting complex, lifelike symptoms and responses [122]. These AI-driven avatars could react dynamically to treatments, providing immediate and realistic feedback. Such advancements will bridge the gap between simulation and real-life clinical encounters, greatly enhancing preparedness for actual patient interactions. The GLLMMs will allow advanced interactivity between multi-sensory modalities such as visual, audio, and haptic. There will be increased ease in synthesizing everything from patients and organs to disease states and radiographic studies.

Medical simulation is heavily invested in scenario-based training, as individuals and teams use computerized mannequins (CMs) or SPs. To some extent, these two scenario-based modalities represent two sides of the same coin: trying to provide a physically evocative semblance of a patient. However, very quickly, these two methodologies will converge for a host of reasons. First, AI allows CMs to be more lifelike and fluidly conversant when queried by scenario participants. Secondly, AI will give mannikins a voice, allowing for high-quality animatronic reactivity in limb movement and facial expression [123]. Several virtual patient platforms are trying to add the dimension of haptic feedback, i.e., palpation, to the IVR experience [124]. Alternatively, mixed reality could combine realistically constructed tissues and surgical instrumentation co-registered and embedded in an IVR. Standardized patients require extensive training and redundancy to meet the needs of a busy simulation program. Some programs have rosters of SPs numbering in the hundreds. It is anticipated that, shortly, the OSCE will be supplanted by a virtual version of it [125]. Artificial intelligence-driven simulations will become more accessible and affordable worldwide, breaking down geographical and financial barriers in medical education. High-quality training could be delivered remotely via cloud-based platforms, making advanced medical education accessible even in resource-limited settings. This democratization of medical training could lead to a more even distribution of healthcare expertise around the world. Future medical simulations will integrate predictive healthcare models, allowing HCPs to simulate and prepare for potential epidemics or patient-specific outcomes. Artificial intelligence could analyze vast amounts of healthcare data to predict trends and simulate future scenarios, such as the outbreak of a new infectious disease, thereby aiding in proactive healthcare planning and response.

## Conclusions

Artificial intelligence will not put trained physicians out of business, but it will change how they do business. Trained physicians are here to stay, but not necessarily in their current numbers in all specialties. Some will be more pressured by AI than others, and in that regard, AI may begin to affect the career choices our students and trainees will make in the coming years. Artificial intelligence will continue to be a disruptive agent in healthcare education. It will represent curricular challenges and demand reconfiguration of many of our educational objectives. It will demand designing, developing, testing, and implementing AI-compatible simulations that can take advantage of the enhanced functionality that AI can bestow on medical education and training. It will make formative simulation more accessible, customizable, realistic, invasive, and pervasive than any educational technology we have ever seen. Finally, there is guarded recognition that AI may also represent an existential threat to humanity itself. As of yet, there are no national standards to regulate the development or propagation of AI, nor are there specific government agencies that are presently positioned to guide as to how we effectively regulate a technology primed to dominate the health and well-being of our citizenry. Preparing ourselves adequately for the challenges of AI now represents the highest priority for the entire ecosystem of healthcare education.
